# Application of machine learning-based multi-sequence MRI radiomics in diagnosing anterior cruciate ligament tears

**DOI:** 10.1186/s13018-024-04602-5

**Published:** 2024-01-31

**Authors:** Qi Cheng, Haoran Lin, Jie Zhao, Xiao Lu, Qiang Wang

**Affiliations:** Department of Orthopedic Surgery, The First Affiliated Hospital of Wannan Medical College, Yijishan Hospital, Wuhu, 241001 Anhui People’s Republic of China

**Keywords:** Anterior cruciate ligament tear, Machine learning, Magnetic resonance imaging, Radiomics

## Abstract

**Objective:**

To compare the diagnostic power among various machine learning algorithms utilizing multi-sequence magnetic resonance imaging (MRI) radiomics in detecting anterior cruciate ligament (ACL) tears. Additionally, this research aimed to create and validate the optimal diagnostic model.

**Methods:**

In this retrospective analysis, 526 patients were included, comprising 178 individuals with ACL tears and 348 with a normal ACL. Radiomics features were derived from multi-sequence MRI scans, encompassing T1-weighted imaging and proton density (PD)-weighted imaging. The process of selecting the most reliable radiomics features involved using interclass correlation coefficient (ICC) testing, t tests, and the least absolute shrinkage and selection operator (LASSO) technique. After the feature selection process, five machine learning classifiers were created. These classifiers comprised logistic regression (LR), support vector machine (SVM), K-nearest neighbors (KNN), light gradient boosting machine (LightGBM), and multilayer perceptron (MLP). A thorough performance evaluation was carried out, utilizing diverse metrics like the area under the receiver operating characteristic curve (ROC), specificity, accuracy, sensitivity positive predictive value, and negative predictive value. The classifier exhibiting the best performance was chosen. Subsequently, three models were developed: the PD model, the T1 model, and the combined model, all based on the optimal classifier. The diagnostic performance of these models was assessed by employing AUC values, calibration curves, and decision curve analysis.

**Results:**

Out of 2032 features, 48 features were selected. The SVM-based multi-sequence radiomics outperformed all others, achieving AUC values of 0.973 and 0.927, sensitivities of 0.933 and 0.857, and specificities of 0.930 and 0.829, in the training and validation cohorts, respectively.

**Conclusion:**

The multi-sequence MRI radiomics model, which is based on machine learning, exhibits exceptional performance in diagnosing ACL tears. It provides valuable insights crucial for the diagnosis and treatment of knee joint injuries, serving as an accurate and objective supplementary diagnostic tool for clinical practitioners.

## Introduction

The anterior cruciate ligament (ACL) is vital for maintaining knee joint stability through the prevention of anterior tibial translation, and preservation of normal knee function [1, 2]. Injuries or severe laxity in the ACL can cause knee joint instability, leading to prominent symptoms and complications, including knee osteoarthritis, as well as meniscal and cartilage injuries [3–5]. Hence, timely and accurate diagnosis, along with early intervention, becomes crucial to restore knee stability and function [6]. While arthroscopic examination is considered the gold standard for diagnosing ACL injuries, it is invasive and involves surgical risks [7, 8]. Conversely, magnetic resonance imaging (MRI) is considered an ideal approach for diagnosing ACL injuries, presenting benefits like high contrast, high resolution, non-invasiveness, and multi-planar imaging. MRI not only offers a clear view of the normal ACL morphology but also provides detailed information on the location and extent of the tear, and other knee joint injuries linked to ACL damage [9, 10]. Nevertheless, diagnosing ACL injuries through MRI often depends on visual assessment by radiologists, a procedure that consumes considerable time and heavily relies on the expertise of the physician in charge. Furthermore, even among experienced radiologists, both inter-observer and intra-observer consistency in interpreting knee MRI scans remains moderately reliable at best [11].

Researchers have started integrating radiomics with machine learning techniques to improve the accuracy and efficiency of ACL injury diagnosis. Radiomics serves as a high-throughput, automated analytical method for clinical imaging data, providing significant assistance in disease diagnosis and prognosis [12–14]. The combination of radiomics and machine learning techniques has shown significant potential in the accurate diagnosis and classification of musculoskeletal disorders. This synergistic effect has enhanced diagnostic efficiency in various tasks, including detecting and characterizing acute joint injuries, chronic pathologies, spinal fractures, degenerative diseases, and tumors [15].

Many studies have explored radiomics-based ACL injury diagnosis [16–19]. Nevertheless, most of these focus on extracting features from a single MRI sequence, which will ignore some important radiomics features. Several studies have demonstrated that models constructed using multi-sequence MRI exhibit a significant performance advantage over their single-sequence counterparts [20–22]. Therefore, we suggest using multi-sequence MRI, as it may contain more valuable information. Furthermore, these studies emphasize deep learning applications, affording limited regard to the potential of traditional machine learning algorithms. Recently, different machine learning classifiers have been compared to determine an optimal machine learning method [23, 24]. Hence, we compared five machine learning algorithms to select the most performant one for model building.

This research aimed to integrate multi-sequence MRI radiomics with machine learning algorithms to extract more valuable radiomics features. The goal was to substantially enhance the diagnostic performance and accuracy in ACL tear diagnosis.

## Materials and methods

### Patients

**Fig. 1:**
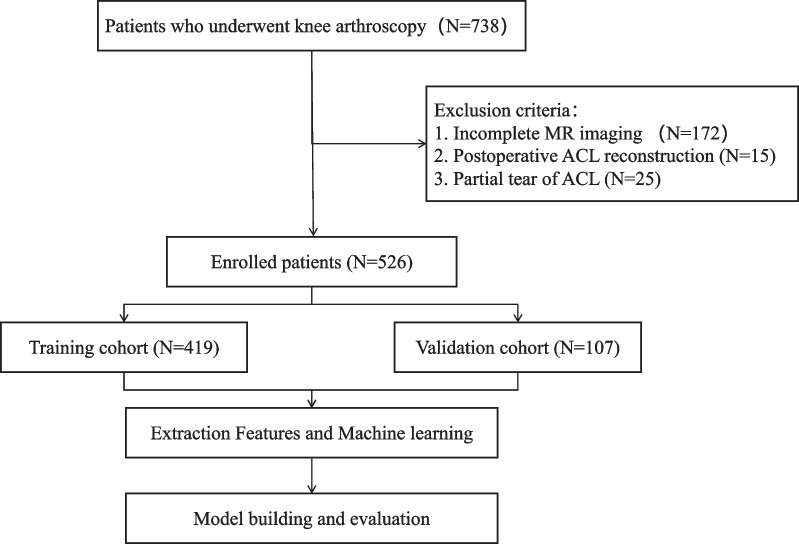
Flowchart detailing the patient recruitment process

Data obtained from knee arthroscopy procedures conducted at the hospital between January 2019 and May 2023 were acquired for this research. Patient records of individuals who underwent knee MRI scans were accessed from the Picture Archiving and Communication System (PACS) at the First Affiliated Hospital of Wannan Medical College, Anhui, China. Knee arthroscopy findings served as the diagnostic reference standard to confirm ACL condition for all patients. The study followed the inclusion and exclusion criteria, as well as the patient recruitment procedure, outlined in Fig. 1. Approval for this retrospective research was granted by the institutional review board, and the need for written informed consent was waived.

### MRI acquisition

For all patients, imaging examinations were conducted using a Siemens Avanto 1.5-T magnetic resonance scanner. The magnetic resonance parameters for image acquisition were configured as follows: T1WI-sag:a field of view measuring 16 cm, an echo time of 11 ms, a repetition time of 400 ms, a slice thickness of 4.0 mm, and a flip angle of 90°; PDWI-sag:a field of view measuring 16 cm, an echo time of 48 ms, a repetition time of 3000 ms, a slice thickness of 4.0 mm, and a flip angle of 150°. In each case, sagittal T1-weighted images (T1WIs) and proton density (PD)-weighted images (PDWIs) with fat suppression were downloaded from the PACS.

### Image segmentation and extraction of radiomics features

**Fig. 2:**
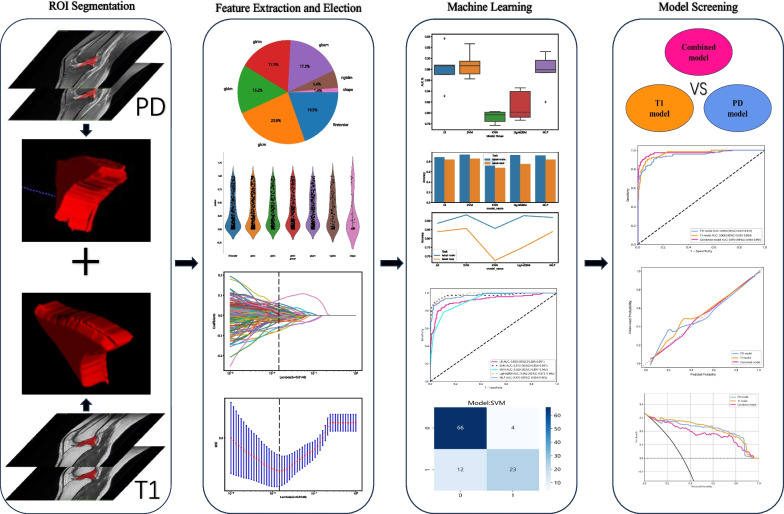
Radiomics workflow diagram

Normalization can reduce the differences caused by different imaging parameters; while, Gaussian filtering can be used to denoise the image [25, 26]. We employed these two methods for image preprocessing to ensure the accuracy of the image data. In this study, manual and independent segmentation of sagittal T1WI and PDWI three-dimensional (3D) region of interest (ROI) was carried out by two radiologists utilizing the ITK-SNAP application (3.8.0; http://www.itksnap.org) [27]. These 3D-ROIs encompassed the entire intercondylar fossa region. Subsequently, the PyRadiomics software package, utilizing Python 3.6, was employed to extract high-throughput radiomics features from the volume of interest of each patient. The extracted radiomics features encompassed shape, first-order, second-order, and higher-order features, with 1016 image-based radiomics features for each sequence in total. Shape features reflect the shape and size of the lesion, such as volume, density, maximum diameter, and surface area. First-order features are also known as histogram features, providing the simplest information level based on the distribution of individual pixel/voxel values within the lesion, without emphasizing their spatial relationship. Second-order features are known as gray-level co-occurrence matrix (GLCM) features, containing more texture information by considering the intensity relationship between adjacent pixel/voxel pairs. High-order features go further by emphasizing the correlation between multiple pixels/voxels, providing complex patterns and texture information. Gray-level size length matrix, neighboring gray-level dependence matrix, gray-level difference matrix, gray-level size zone matrix, and gray-level distance zone matrix are some examples of high-order features [12]. Interclass correlation coefficients (ICCs) were computed and features with ICC values < 0.75 were excluded to guarantee the stability and accuracy of the radiomics features. The workflow of the proposed approach in this research is depicted in Fig. 2.

### Features selection

Radiomics features extracted from T1WIs and PDWIs were standardized utilizing the z-score technique. Subsequently, a t test was applied to evaluate the entire array of radiomics features, retaining only those with P-values < 0.05. Further analysis involved the computation of Spearman correlation coefficients to assess the relationships between features. In instances where any two correlation coefficients exceeded 0.9, only one feature was retained. Subsequently, using an 8:2 ratio, all samples were randomly classified into training and validation sets. A greedy recursive feature elimination strategy was implemented, eliminating the feature with the highest redundancy in the current set at each step. The least absolute shrinkage and selection operator (LASSO) regression model, coupled with tenfold cross-validation, was utilized in the training set for feature selection [28]. Features with non-zero coefficients were subsequently chosen and employed to train the classifier.

### Selection of machine learning classifier

A comparative analysis of five machine learning classifiers, namely logistic regression (LR), support vector machines (SVM), K-nearest neighbors (KNN), light gradient boosting machine (LightGBM), and multilayer perceptron (MLP), was carried out to identify the optimal classifier for the task. During the modeling process, each classifier was carefully optimized and tuned with the aim of maximizing diagnostic performance. For the LR algorithm, the optimal hyperparameters were C = 1, max_iter = 100, and penalty = L2. For the SVM algorithm, the optimal hyperparameters were C = 1 and kernel = rbf. For the KNN algorithm, the optimal hyperparameters were n_neighbours = 4 and weights = 'uniform'. For the LightGBM algorithm, the optimal hyperparameters were boosting_type = 'gbdt' and learning_rate = 0.001. And for the MLP algorithm, the optimal hyperparameters were activation = 'relu' and max_iter = 100. In the training set, the diagnostic performance of various classifiers was assessed by evaluating the area under the curve (AUC), accuracy, sensitivity, specificity, positive and negative predictive values, recall, and F1 score. Calibration curves were constructed to determine the consistency across the predicted and actual outcomes. Furthermore, a comprehensive evaluation of the clinical applicability of the models was conducted utilizing decision curve analysis (DCA). Using these findings, the classifier with the most favorable overall performance was chosen.

### Construction of the machine learning model

Following the selection of the most suitable classifier, it was then utilized to construct three models: the T1 model, the PD model, and the combined model. Each model underwent individual performance assessment, which included evaluating sensitivity, specificity, accuracy, and the area under the receiver operating characteristic (ROC) curve. These evaluation metrics allowed for a comparative analysis of the classification abilities and predictive accuracy of the different models. Ultimately, the model that demonstrated the most exceptional performance in the training set was chosen as the final model.

### Statistical analysis

All statistical analyses, normalization, feature selection, and model building were performed using Python 3.7.0, NumPy, Matplotlib, Scikit-learn, and Pyradiomics software packages [29–31]. The measurement data were tested for normality, and those that conformed to a normal distribution were expressed as (mean ± standard deviation), and the independent samples t test was used for comparison between two groups; for measurement data that did not conform to a normal distribution, Mann–Whitney U test was used for comparison between two groups. Statistical significance was established at P < 0.05 (two-sided).

## Results

### Patient characteristics

In this research, 526 patients who underwent knee arthroscopy were included. Among them, 178 were diagnosed with ACL tears; while, 348 patients had intact ACLs. A total of 278 and 248 patients were males and females, respectively. The average age of the baseline was 43.37 ± 14.60 years. The 526 patients included 262 left knees and 264 right knees. Using an 8:2 ratio, the individuals were classified randomly into training (N = 419) and validation (N = 107) sets.

### Radiomics feature extraction and selection

**Fig. 3:**
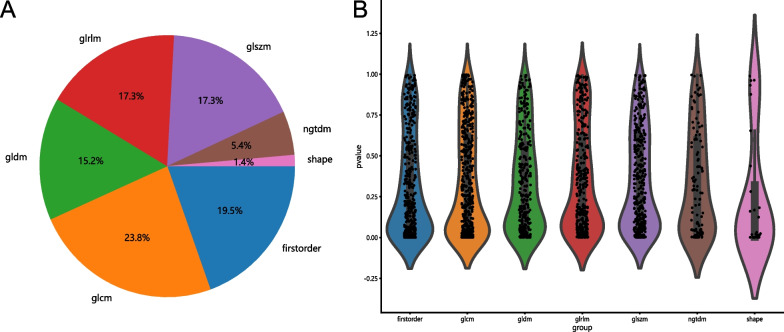
Proportion, distribution, and P value of different radiomics features

Initially, 2032 radiomic features were extracted, out of which 1942 features with ICC values > 0.75 were retained. Subsequent t tests identified 542 features with a significance level of P < 0.05. Figure 3 illustrates the distribution of these radiomic features and their corresponding P values. Spearman correlation coefficients were calculated among these features to address the issue of high intercorrelation between features. This led to retaining a single feature from each pair with a correlation coefficient exceeding 0.9. Consequently, 209 features were ultimately retained.

**Fig. 4:**
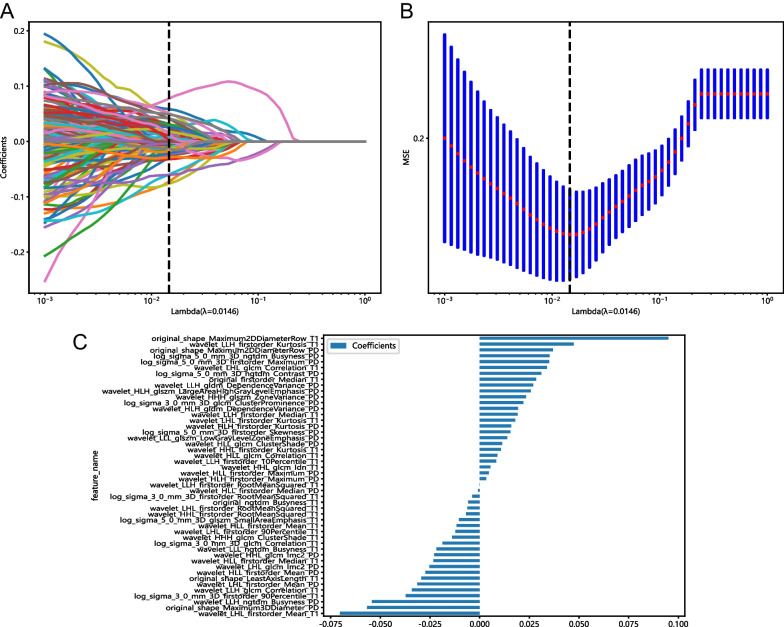
**A** Feature reduction and selection using least absolute shrinkage and selection operator (LASSO) based on the minimum log (λ) with tenfold cross-validation. **B** Lambda values correlated with the number of features. **C** Feature weights following feature selection using the LASSO algorithm

Using the LASSO classifier, 48 optimal radiomic features (26 from T1WI and 22 from PDWI) were selected. Figure 4 have illustrated the outcomes of the tenfold cross-validation regression, displaying the final selection of radiomic features along with their corresponding coefficients. Among the features, first-order features and shape features with higher correlation coefficients are considered the most significant ones. First-order features are obtained through statistical analysis of image pixel values or grayscale levels, which describes the distribution and frequency of pixels in the image. For example, the MRI images after ACL tear showed changes in signal intensity, leading to differences in first-order features. On the other hand, shape features were used to describe the lesion's area, volume, perimeter, irregularity, and density. The tears of ACL, its discontinuity and shape changes would results in higher correlation coefficients for shape features. Features and corresponding coefficients were put in the Additional file 1: Supplementary Table S1.

### Comparison of various machine learning classifiers

**Fig. 5:**
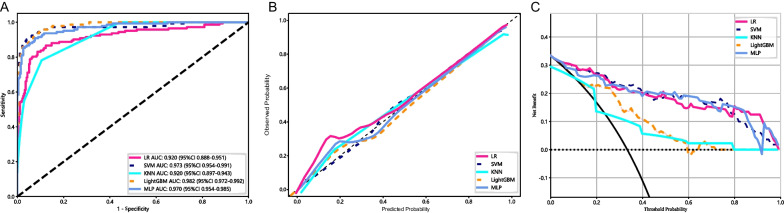
Performance evaluation of various classifiers **A** Receiver operating characteristics curves. **B** Calibration curves. **C** Decision curves

A comparative evaluation of the diagnostic performance of five machine learning classifiers was conducted, with each classifier trained using its respective optimal hyperparameters on the training set. Figure 5 displays the ROC, calibration, and DCA curves for all classifiers; while, Table 1 presents the AUC, accuracy, sensitivity, specificity, positive and negative predictive values, recall, and F1 score for each machine learning classifier.

**Table 1:** Performance metrics of different classifiers in the training set

Classifier	AUC	Accuracy	Sensitivity	Specificity	PPV	NPV	Recall	F1 score
LR	0.920	0.885	0.831	0.913	0.831	0.913	0.831	0.831
SVM	0.973	**0.933**	**0.930**	**0.935**	**0.880**	**0.963**	**0.930**	**0.904**
KNN	0.920	0.857	0.782	0.895	0.793	0.889	0.782	0.787
LightGBM	**0.982**	0.928	**0.930**	0.928	0.868	0.962	**0.930**	0.898
MLP	0.970	0.919	0.908	0.924	0.860	0.952	0.908	0.884

The bold values are the optimal values of each quantitative indicator

The respective AUC values for LR, SVM, KNN, LightGBM, and MLP were 0.920, 0.973, 0.920, 0.982, and 0.970. LightGBM exhibited the highest AUC of 0.982 (95% confidence interval [CI]: 0.972–0.992). However, SVM demonstrated superior performance across various metrics, encompassing accuracy (93.3%), sensitivity (93.0%), specificity (93.5%), positive predictive value (88.0%), and negative predictive value (96.3%), alongside an AUC of 0.973 (95% CI: 0.954–0.991). Moreover, the calibration curve for SVM implied strong agreement between model predictions and observed outcomes (Fig. 5B). Furthermore, the DCA indicated that the net benefit derived from the SVM classifier exceeded that of the other four classifiers (Fig. 5C). The y-axis represents net benefit, and the x-axis represents threshold probability. Across the entire range of threshold probabilities, SVM demonstrates higher overall net benefit in both full intervention (black diagonal line) and no intervention (dashed line).

Consequently, after a thorough performance evaluation of the training set, SVM emerged as the optimal classifier.

### Model selection

**Fig. 6:**
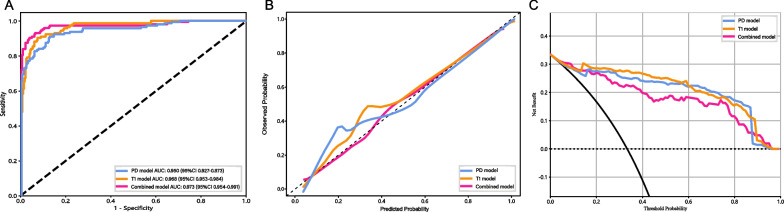
Performance evaluation of different models **A** Receiver operating characteristics curves. **B** Calibration curves. **C** Decision curves

Three models were developed utilizing the SVM classifier to assess the influence of different feature sets. A quantitative comparison of the diagnostic performance of these models was then conducted utilizing the test set, with the results detailed in Table 2. Additionally, Fig. 6 presents the corresponding ROC curves, calibration curves, and DCA curves.

**Table 2:** Performance metrics of various models in both the training and validation sets

Model	Cohort	AUC (95% CI)	Accuracy	Sensitivity	Specificity	PPV	NPV
Combined	Train	0.973(0.954–0.991)	0.933	0.930	0.935	0.880	0.963
	Validation	0.927(0.878–0.976)	0.857	0.829	0.871	0.763	0.910
T1	Train	0.968(0.953–0.984)	0.919	0.902	0.928	0.866	0.949
	Validation	0.878(0.804–0.951)	0.848	0.800	0.871	0.757	0.897
PD	Train	0.950(0.927–0.973)	0.886	0.909	0.874	0.788	0.949
	Validation	0.904(0.847–0.962)	0.829	0.857	0.814	0.698	0.919

The respective AUC values for the T1 model and PD model were 0.968 and 0.950. However, the combined model demonstrated a superior AUC in comparison with the T1 and PD models. Specifically, in the training set, the combined model achieved an AUC of 0.973 (95% CI: 0.954–0.991), with accuracy, sensitivity, specificity, positive and negative predictive values recorded at 93.3%, 93.0%, 93.5%, 88.0%, and 96.3%, respectively. Furthermore, in the validation set, the combined model yielded the highest AUC of 0.927, along with accuracy, sensitivity, specificity, and positive and negative predictive values recorded at 93.3%, 93.0%, 93.5%, 88.0%, and 96.3%, respectively.

The ROC curves of the three models within the training set are depicted in Fig. 6A, highlighting the superior AUC of the combined model in comparison with the other two models. This superiority is further supported by the calibration curve (Fig. 6B), emphasizing the alignment of predictions from the combined model with observed values, signifying a higher level of consistency than the other models. Furthermore, the DCA results (Fig. 6C) indicated that the combined model provided a greater net benefit compared to the other models. These collective findings indicate that the diagnostic performance of the combined model surpassed that of the T1 and PD models. Consequently, the combined model, based on SVM, was chosen as the final and optimal model.

### Model validation

**Fig. 7:**
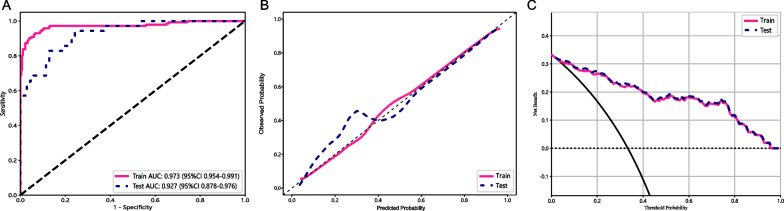
Model validation results **A** The receiver operating characteristic curves for the training and validation sets. **B** Calibration curves for these two sets. **C** Decision curves for these two sets

Validation was performed using a validation dataset for further assessment of the clinical usage of the hybrid model. Figure 7 presents the ROC curves, calibration curves, and DCA curves of the hybrid model based on SVM. The respective AUC values for the training and validation sets were 0.973 (95% CI 0.954–0.991) and 0.927 (95% CI 0.878–0.976). The calibration curves verified the excellent fitting performance of the hybrid model in both the training and validation sets. Moreover, the DCA curves indicated that the hybrid model displayed favorable clinical utility in both of these sets.

## Discussion

The longer the delay between ACL rupture and reconstruction, the greater the risk of meniscal and/or chondral injuries may be [32, 33]. Early intervention plays a crucial role in minimizing the damage to meniscal and chondral tissue following an ACL injury. This not only reduces pain but also contributes to faster rehabilitation, improves quality of life, and enables patients to return to normal life and sports activities as soon as possible [34]. This study utilized manually annotated multi-sequence knee joint MRI ROIs and skillfully extracted specific radiomic features. Robust features were selected; while, redundant ones were excluded using LASSO regression. After this, 48 features were finally kept. Among them, there are 23 first-order features, which are the simplest representations of information in the image. They are computed from the grayscale histogram of the image and are used to describe the distribution and frequency of pixels with specific grayscale intensities within the ROI. After ACL tear, the signal intensity of ACL decreases in the sagittal slice of T1WI; while, it increases in the sagittal slice of PDWI. As the signal changes, the pixels within the ROI also change accordingly, making first-order features crucial. Furthermore, GLCM features can provide more information than the image histogram and contain information about the spatial relationship between pixels pairs with similar or specific intensities [35]. Therefore, we selected 10 GLCM features with non-zero coefficients. In addition, we also screened out 11 high-order features and 4 shape features. High-order features provide richer information by emphasizing correlations between multiple pixels/voxels, providing complex patterns and texture information. After ACL tears, its shape changes, and it appears as swelling and thickening of the ligament on MRI. The diagnostic performance of an MRI radiomic model was compared across five machine learning algorithms in the context of ACL tear diagnosis.

After a comprehensive examination of various machine learning methods, the SVM-based radiomic model exhibited superior diagnostic performance. This model achieved an AUC of 0.973 (95% CI 0.954–0.991) and demonstrated exceptional accuracy at 93.3%, along with high sensitivity (93.0%), specificity (93.5%), positive predictive value (88.0%), and negative predictive value (96.3%).

Previous research predominantly employed deep learning algorithms for diagnosing ACL tears, In 2018, the first relevant study was published, deep convolutional neural network (DCNN) to fully automate the detection of ACL tears. This DCNN achieved a sensitivity of 76%, specificity of 97%, and an AUC of 0.97 for ACL tear detection [16]. Another study applied deep learning architecture to ACL lesion detection and achieved a sensitivity of 96% and specificity of 96%, and AUC of 0.98 [17]. Germann et al. developed a deep learning model for ACL tear detection, which showed a sensitivity of 0.99, specificity of 0.94, and an AUC of 0.97 [18]. These studies demonstrating the potential of radiomics and deep learning in the realm of ACL tears diagnosis. However, although deep learning techniques are considered cutting-edge for image classification, carry inherent complexity that often renders the interpretation of decision outcomes challenging, thus posing issues related to interpretability [36]. In contrast, machine learning models are generally more interpretable, and the decision process of the model can be explained using feature weights and other methods [37].

Hence, in this research, the performance of five machine learning algorithms (LR, SVM, KNN, LightGBM, and MLP) was compared. These algorithms were meticulously assessed using various metrics, including AUC, accuracy, sensitivity, specificity, positive and negative predictive values, recall, and F1 score. Eventually, SVM was chosen as the optimal algorithm. SVM operates by identifying the optimal hyperplane within the data space to effectively segregate samples belonging to different classes [38, 39]. Previous studies, such as the one conducted by Chen et al. [40], also reported the efficacy of SVM classifiers in diagnosing ACL tears. However, his study solely compared the performance of the random forest (RF) and SVM classifiers. Different machine learning methods yield diverse classification outcomes. Consequently, this comprehensive analysis of multiple machine learning algorithms aimed to identify the most valuable and stable algorithm in ACL tear diagnosis.

Until now, previous research in this domain has predominantly relied on a single sequence for radiomic data extraction, resulting in a constrained scope of feature extraction [19, 41, 42]. The amalgamation of multiple sequences presents an opportunity to encompass a more extensive range of information, thereby facilitating a more comprehensive description of the characteristics inherent to the ROIs [43]. Liu et al. also demonstrated in their study that using multiple-sequence MRI can extract more radiomic features, thereby improving the sensitivity and specificity of the model [17]. Therefore, this study compared the performance of single-sequence and multi-sequence models. Interestingly, the findings reaffirmed the superiority of the multi-sequence model, highlighting the presence of complementary information among different MRI sequences. This underscores the potential for a more precise diagnosis of ACL tears through the use of multiple MRI sequences, offering a more comprehensive description of radiomic features.

The findings of this study underscore the capacity of machine learning algorithms based on multiple-sequence MRI to accurately identify ACL tears. Diagnosing ACL tears might not be a challenge for expert musculoskeletal radiologists and sports medicine physicians. However, this study carries significant utility for non-specialist radiologists and non-sports medicine physicians. Particularly in rural areas where access to specialized radiology experts or professional radiological interpretation might be constrained, this study offers a valuable reference for their “second opinion”.

Our model can assist doctors in making more accurate and timely diagnoses, enabling prompt intervention and effective management. This not only reduces pain and further damage but also helps improve patients’ quality of life, accelerates the recovery process, and assists them in returning to normal life and sports activities as soon as possible.

Nevertheless, it is imperative to acknowledge certain limitations of this research. First, it represents a retrospective investigation founded on relatively small sample size, underscoring the need for larger datasets to enhance the reliability and clinical applicability of radiomics research. Second, this study only extracted radiomic information from the sagittal plane, although there might have been radiomic features potentially extractable from axial and coronal plane MRI scans. Thus, future research endeavors should further explore the feasibility of harnessing images from different planes to extract comprehensive radiomic information to enhance diagnostic performance and accuracy. Thirdly, this study is a single-center retrospective study without external validation, which may potentially impact the reliability and generalizability of the model. We will strive to obtain external validation datasets in future research to address this issue.

## Conclusion

This study showcased exceptional diagnostic performance by utilizing multi-sequence MRI to extract radiomic features and constructing a model for identifying ACL tears using the SVM classifier. This research offers valuable insights for diagnosing and treating knee joint injuries, providing clinical physicians with an objective and accurate auxiliary diagnostic tool.

## References

[1] Veltri DM, Deng XH, Torzilli PA, Warren RF, Maynard MJ (1995). The role of the cruciate and posterolateral ligaments in stability of the knee. A biomechanical study. Am J Sports Med.

[2] Lam MH, Fong DT, Yung P, Ho EP, Chan WY, Chan KM (2009). Knee stability assessment on anterior cruciate ligament injury: Clinical and biomechanical approaches. Sports Med Arthrosc Rehabil Ther Technol.

[3] NegahiShirazi A, Chrzanowski W, Khademhosseini A, Dehghani F (2015). Anterior cruciate ligament: structure, injuries and regenerative treatments. Adv Exp Med Biol.

[4] Kwee RM, Hafezi-Nejad N, Roemer FW, Zikria BA, Hunter DJ, Guermazi A, Demehri S (2018). Association of mucoid degeneration of the anterior cruciate ligament at MR imaging with medial tibiofemoral osteoarthritis progression at radiography: data from the osteoarthritis initiative. Radiology.

[5] Nelson F, Billinghurst RC, Pidoux I, Reiner A, Langworthy M, McDermott M, Malogne T, Sitler DF, Kilambi NR, Lenczner E (2006). Early post-traumatic osteoarthritis-like changes in human articular cartilage following rupture of the anterior cruciate ligament. Osteoarthritis Cartilage.

[6] Dold AP, Swensen S, Strauss E, Alaia M (2017). The posteromedial corner of the knee: anatomy, pathology, and management strategies. J Am Acad Orthop Surg.

[7] Shantanu K, Singh S, Srivastava S, Saroj AK (2021). The validation of clinical examination and MRI as a diagnostic tool for cruciate ligaments and meniscus injuries of the knee against diagnostic arthroscopy. Cureus.

[8] Bari AA, Kashikar SV, Lakhkar BN, Ahsan MS (2014). Evaluation of MRI versus arthroscopy in anterior cruciate ligament and meniscal injuries. JCDR.

[9] Li Z, Ren S, Zhou R, Jiang X, You T, Li C, Zhang W (2021). Deep learning-based magnetic resonance imaging image features for diagnosis of anterior cruciate ligament injury. J Healthc Eng.

[10] Li K, Du J, Huang LX, Ni L, Liu T, Yang HL (2017). The diagnostic accuracy of magnetic resonance imaging for anterior cruciate ligament injury in comparison to arthroscopy: a meta-analysis. Sci Rep.

[11] Quatman CE, Hettrich CM, Schmitt LC, Spindler KP (2011). The clinical utility and diagnostic performance of magnetic resonance imaging for identification of early and advanced knee osteoarthritis: a systematic review. Am J Sports Med.

[12] Gillies RJ, Kinahan PE, Hricak H (2016). Radiomics: images are more than pictures, they are data. Radiology.

[13] Mahmud M, Kaiser MS, McGinnity TM, Hussain A (2021). Deep learning in mining biological data. Cogn Comput.

[14] Lambin P, Rios-Velazquez E, Leijenaar R, Carvalho S, van Stiphout RG, Granton P, Zegers CM, Gillies R, Boellard R, Dekker A (2012). Radiomics: extracting more information from medical images using advanced feature analysis. Eur J Cancer (Oxford, England: 1990).

[15] Fritz B, Yi PH, Kijowski R, Fritz J (2023). Radiomics and deep learning for disease detection in musculoskeletal radiology: an overview of novel MRI- and CT-based approaches. Invest Radiol.

[16] Bien N, Rajpurkar P, Ball RL, Irvin J, Park A, Jones E, Bereket M, Patel BN, Yeom KW, Shpanskaya K (2018). Deep-learning-assisted diagnosis for knee magnetic resonance imaging: development and retrospective validation of MRNet. PLoS Med.

[17] Liu F, Guan B, Zhou Z, Samsonov A, Rosas H, Lian K, Sharma R, Kanarek A, Kim J, Guermazi A (2019). Fully automated diagnosis of anterior cruciate ligament tears on knee MR images by using deep learning. Radiol Artif Intell.

[18] Germann C, Marbach G, Civardi F, Fucentese SF, Fritz J, Sutter R, Pfirrmann CWA, Fritz B (2020). Deep convolutional neural network-based diagnosis of anterior cruciate ligament tears: performance comparison of homogenous versus heterogeneous knee MRI cohorts with different pulse sequence protocols and 1.5-T and 3-T magnetic field strengths. Invest Radiol.

[19] Chang PD, Wong TT, Rasiej MJ (2019). Deep learning for detection of complete anterior cruciate ligament tear. J Digit Imaging.

[20] Wang G, He L, Yuan C, Huang Y, Liu Z, Liang C (2018). Pretreatment MR imaging radiomics signatures for response prediction to induction chemotherapy in patients with nasopharyngeal carcinoma. Eur J Radiol.

[21] Wu M, Xu W, Fei Y, Li Y, Yuan J, Qiu L, Zhang Y, Chen G, Cheng Y, Cao Y (2023). MRI-based clinical radiomics nomogram may predict the early response after concurrent chemoradiotherapy in locally advanced nasopharyngeal carcinoma. Front Oncol.

[22] Tsuchiya M, Masui T, Terauchi K, Yamada T, Katyayama M, Ichikawa S, Noda Y, Goshima S (2022). MRI-based radiomics analysis for differentiating phyllodes tumors of the breast from fibroadenomas. Eur Radiol.

[23] Nakagawa M, Nakaura T, Namimoto T, Iyama Y, Kidoh M, Hirata K, Nagayama Y, Yuki H, Oda S, Utsunomiya D (2019). Machine learning to differentiate T2-weighted hyperintense uterine leiomyomas from uterine sarcomas by utilizing multiparametric magnetic resonance quantitative imaging features. Acad Radiol.

[24] Shu Z, Mao D, Song Q, Xu Y, Pang P, Zhang Y (2022). Multiparameter MRI-based radiomics for preoperative prediction of extramural venous invasion in rectal cancer. Eur Radiol.

[25] Rezaeijo SM, Chegeni N, Baghaei Naeini F, Makris D, Bakas S. Within-modality synthesis and novel radiomic evaluation of brain MRI scans. Cancers (Basel). 2023;15(14). 10.3390/cancers15143565 PMC10377568 37509228

[26] Khanfari H, Mehranfar S, Cheki M, Mohammadi Sadr M, Moniri S, Heydarheydari S, Rezaeijo SM (2023). Exploring the efficacy of multi-flavored feature extraction with radiomics and deep features for prostate cancer grading on mpMRI. BMC Med Imaging.

[27] Yushkevich PA, Yang G, Gerig G (2016). ITK-SNAP: an interactive tool for semi-automatic segmentation of multi-modality biomedical images. Annual International Conference of the IEEE Engineering in Medicine and Biology Society IEEE Engineering in Medicine and Biology Society Annual International Conference.

[28] Tibshirani R (1997). The lasso method for variable selection in the Cox model. Stat Med.

[29] Hosseinzadeh M, Gorji A, Fathi Jouzdani A, Rezaeijo SM, Rahmim A, Salmanpour MR. Prediction of cognitive decline in Parkinson's disease using clinical and DAT SPECT imaging features, and hybrid machine learning systems. Diagnostics (Basel). 2023;13(10). 10.3390/diagnostics13101691 PMC10217464 37238175

[30] Heydarheydari S, Birgani MJT, Rezaeijo SM (2023). Auto-segmentation of head and neck tumors in positron emission tomography images using non-local means and morphological frameworks. Pol J Radiol.

[31] Bridge CP, Gorman C, Pieper S, Doyle SW, Lennerz JK, Kalpathy-Cramer J, Clunie DA, Fedorov AY, Herrmann MD (2022). Highdicom: a python library for standardized encoding of image annotations and machine learning model outputs in pathology and radiology. J Digit Imaging.

[32] Jones HP, Appleyard RC, Mahajan S, Murrell GA (2003). Meniscal and chondral loss in the anterior cruciate ligament injured knee. Sports Med.

[33] Gregory T, Landreau P (2008). Meniscus and cartilaginous lesions. Influence of the delay between ACL injury and ligament reconstruction in 40-year-old patients. Rev Chir Orthop Reparatrice Appar Mot.

[34] Tayton E, Verma R, Higgins B, Gosal H (2009). A correlation of time with meniscal tears in anterior cruciate ligament deficiency: stratifying the risk of surgical delay. Knee Surg Sports Traumatol Arthrosc.

[35] AbbasianArdakani A, Bureau NJ, Ciaccio EJ, Acharya UR (2022). Interpretation of radiomics features-A pictorial review. Comput Methods Programs Biomed.

[36] Tran A, Lassalle L, Zille P, Guillin R, Pluot E, Adam C, Charachon M, Brat H, Wallaert M, d'Assignies G (2022). Deep learning to detect anterior cruciate ligament tear on knee MRI: multi-continental external validation. Eur Radiol.

[37] Choi RY, Coyner AS, Kalpathy-Cramer J, Chiang MF, Campbell JP (2020). Introduction to machine learning, neural networks, and deep learning. Transl Vis Sci Technol.

[38] Zhou S (2022). Sparse SVM for sufficient data reduction. IEEE Trans Pattern Anal Mach Intell.

[39] Tsai CA, Chang YJ. Efficient selection of Gaussian Kernel SVM parameters for imbalanced data. Genes. 2023, 14(3). 10.3390/genes14030583 PMC10048125 36980852

[40] Chen DS, Wang TF, Zhu JW, Zhu B, Wang ZL, Cao JG, Feng CH, Zhao JW (2021). A novel application of unsupervised machine learning and supervised machine learning-derived radiomics in anterior cruciate ligament rupture. Risk Manag Healthc Policy.

[41] Zhang L, Li M, Zhou Y, Lu G, Zhou Q (2020). Deep learning approach for anterior cruciate ligament lesion detection: evaluation of diagnostic performance using arthroscopy as the reference standard. JMRI.

[42] Namiri NK, Flament I, Astuto B, Shah R, Tibrewala R, Caliva F, Link TM, Pedoia V, Majumdar S (2020). Deep learning for hierarchical severity staging of anterior cruciate ligament injuries from MRI. Radiol Artif Intellig.

[43] Wei L, Osman S, Hatt M, El Naqa I (2019). Machine learning for radiomics-based multimodality and multiparametric modeling. Q J Nuclear Med Mol Imaging.

